# Effect of Endogenous and Exogenous Butyric Acid on Butanol Production From CO by Enriched *Clostridia*

**DOI:** 10.3389/fbioe.2022.828316

**Published:** 2022-02-16

**Authors:** Yaxue He, Piet N. L. Lens, María C. Veiga, Christian Kennes

**Affiliations:** ^1^ Chemical Engineering Laboratory, Faculty of Sciences and Center for Advanced Scientific Research (CICA), BIOENGIN Group, University of La Coruña (UDC), A Coruña, Spain; ^2^ National University of Ireland Galway, Galway, Ireland

**Keywords:** butanol, carbon monoxide, butyric acid, glucose, syngas, *Clostridia*

## Abstract

Butanol is a potential renewable fuel. To increase the selectivity for butanol during CO fermentation, exogenous acetic acid and ethanol, exogenous butyric acid or endogenous butyric acid from glucose fermentation have been investigated using CO as reducing power, with a highly enriched *Clostridium* sludge. Addition of 3.2 g/L exogenous butyric acid led to the highest 1.9 g/L butanol concentration with a conversion efficiency of 67%. With exogenous acetate and ethanol supply, the butanol concentration reached 1.6 g/L at the end of the incubation. However, the presence of acetic acid and ethanol favoured butanol production to 2.6 g/L from exogenous butyric acid by the enriched sludge. Finally, exogenous 14 g/L butyric acid yielded the highest butanol production of 3.4 g/L, which was also among the highest butanol concentration from CO/syngas fermentation reported so far. CO addition triggered butanol production from endogenous butyric acid (produced from glucose, Glucose + N_2_) with as high as 58.6% conversion efficiency and 62.1% butanol yield. However, no efficient butanol production was found from glucose and CO co-fermentation (Glucose + CO), although a similar amount of endogenous butyric acid was produced compared to Glucose + N_2_. The *Clostridium* genus occupied a relative abundance as high as 82% from the initial inoculum, while the *Clostridia* and *Bacilli* classes were both enriched and dominated in Glucose + N_2_ and Glucose + CO incubations. This study shows that the supply of butyric acid is a possible strategy for enhancing butanol production by CO fed anaerobic sludge, either via exogenous butyric acid, or via endogenous production by sugar fermentation.

**GRAPHICAL ABSTRACT:**
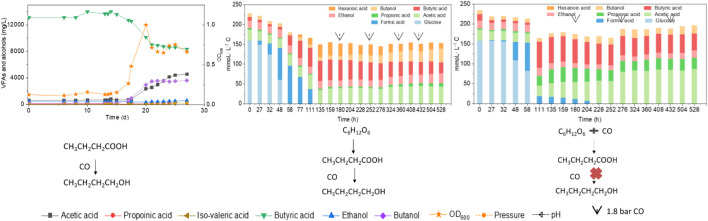
Effect of endogenous and exogenous butyric acid on butanol production from CO by enriched Clostridia.

## Highlights

• 3.4 g/L butanol was produced as highest concentration from CO and butyric acid.
• CO triggered butanol production from endogenous butyric acid at 62.1% butanol yield.
• Acetic acid and ethanol favoured butyric acid to butanol with CO as reducing power.
• *Clostridia* and *Bacilli* classes were dominant with CO, glucose and in co-fermentation.

## 1 Introduction

Carbon monoxide (CO) is one of the main components in some industrial gas emissions such as steel plants and gasification of biomass or municipal solid waste (Yu et al., 2015). Syngas (CO, CO_2_, H_2_) fermentation, which combines syngas and microorganisms together for the production of valuable chemicals, including butanol, has received increasing attention over the last decade (Mohammadi et al., 2011; Tang et al., 2021). Butanol (butyl alcohol and 1-butanol, C_4_H_9_OH) is an alternative liquid fuel because of its similar characteristics to gasoline. Thus, it can be used directly in any gasoline engine without modification and/or substitution, thus gaining more value than ethanol as a biofuel (Lee et al., 2008; Knoshaug and Zhang, 2009). CO fermentation has the advantage of using non-food feedstocks compared to traditional fermentation (Devarapalli and Atiyeh, 2015). In addition, CO is present in off gas of the steel industry, thus this cheap gas substrate can make syngas-based butanol production more economical.

The conversion of CO follows the Wood–Ljungdahl pathway (WLP) to synthesize acetyl-CoA, that can be further converted to acetic acid, biomass and ethanol (Fernández-Naveira et al., 2017a; Teixeira et al., 2018). Several microorganisms convert CO/syngas to ethanol, including *Acetobacterium woodii, Clostridium ljungdahlii, Clostridium autoethanogenum, Clostridium carboxidivorans* and *Butyribacterium methylotrophicum*, but very few can produce higher alcohols (Liu et al., 2014a; Fernández-Naveira et al., 2017a; Cheng et al., 2019). Pure *Clostridium* strains hardly produced butanol at concentrations higher than 2.7 g/L from syngas or CO (Fernández-Naveira et al., 2016). Although inhibitory butanol production at high concentrations is observed in pure cultures, CO conversion to butanol by a broad range of acetogenic mixed cultures has received little attention (Alves et al., 2013). Mixed cultures have the advantage of no need of sterilization and resistance to unfavorable environmental conditions, such as their tolerance to a wide pH range for alcohol production. This enables an easier implementation at large scale compared to pure cultures (Liu et al., 2020).

Acid production in the acidogenic stage is necessary and enhances solvent production in the solventogenic stage (Worden et al., 1991; Xu et al., 2020). Exogenous butyric acid has been used for enhancing butanol production in acetone-butanol-ethanol (ABE) fermentation from organic carbon (Sabra et al., 2014; Luo et al., 2015; Gao et al., 2016; Luo et al., 2017; Munch et al., 2020), but in CO/syngas fermentation this has been seldom reported although CO is a potential reductant for the reduction of butyric acid to butanol. Therefore, one appealing way for enhancing butanol production is first to induce butyric acid production, either endogenously produced or exogenously supplied. Then, butyric acid could be further used for butanol production through butyryl-CoA in the WLP (Fernández-Naveira et al., 2017a).

In addition, acetic acid can be converted into the higher added value compound butyric acid through carbon chain elongation in which ethanol can be used as electron donor promoting the reverse β-oxidation pathway (Baleeiro et al., 2019). Supplied acetic acid and ethanol can be converted to butyric acid by some mid chain acid producers, then, endogenous butyric acid can be converted to butanol by solventogenic acetogens. Some co-fermentation examples have been studied in co-cultures, such as acetogenic *Clostridium kluyveri* and solventogenic *C. autoethanogenum* (Diender et al., 2016) or *C. kluyveri* and solventogenic *C. aceticum* (Fernández-Blanco et al., 2022). The addition of acetate also prevented strain degeneration in *Clostridium* spp. (Chen and Blaschek, 1999).

Endogenous butyric acid production from sugar fermentation could be an alternative source for butyric acid and enhanced butanol production as well (Munch et al., 2020). Glucose, as the typical carbohydrate for synthetic carbohydrate rich wastewater can be converted to butyric acid by *Clostridium* spp., such as *C. carboxidivorans* (Fernández-Naveira et al., 2017b). The metabolism, intermediates and end products may change in the glucose and CO co-fermentation as different reducing equivalents are produced from glucose (via glycolysis) and CO (via the carbon monoxide dehydrogenase (CODH) enzyme) (Sabra et al., 2016). A few reports focused on CO and glucose co-fermentation for methane and hydrogen production, but not for acids and alcohol production (Liu et al., 2020). Additionally, butanol production from co-fermentation of CO/syngas and glucose by mixed cultures is an attractive and economical process (Baleeiro et al., 2019).

To date, selective butanol production from CO and exogenous acids and ethanol on butanol selectivity by mixed cultures has not yet been reported. This research addressed enhanced butanol production by enriched sludge with dominant populations of *Clostridium* spp. using CO as reducing power. The study evaluated different strategies to increase the butyric acid concentration, i.e., via exogenous acetic acid and ethanol supply as well as exogenous and endogenous butyric acid supply. The research further investigated the microbial community and identified the dominant species regulating butanol production via endogenous butyric acid under solely glucose fermentation followed by CO addition (Glucose + N_2_) and co-fermentation of glucose and CO (Glucose + CO) by the enriched sludge.

## 2 Materials and Methods

### 2.1 Source of Inoculum

The inoculum was obtained from a CO fed enriched sludge after six successive biomass transfers described in detail in He et al. (2021a), He et al. (2022). The inoculum contained 81% of *Clostridium* genus reaching an ethanol production of as high as 11.8 g/L with only minor accumulation of acetic acid between pH 6.45 and 4.95. The inoculum could convert exogenous butyric acid into butanol using CO as the reducing power (He et al., 2021a).

### 2.2 Medium Composition

The culture medium was prepared as described previously (He et al., 2022). A 1 L culture medium was prepared according to Stams et al. (1993) and modified as follows: 408 mg/L KH_2_PO_4_, 534 mg/L Na_2_HPO_4_·2H_2_O, 300 mg/L NH_4_Cl, 300 mg/L NaCl, 100 mg/L MgCl_2_·6H_2_O, 110 mg/L CaCl_2_·2H_2_O; 1 ml trace metal and 1 ml vitamin stock solution (Stams et al., 1993). Once prepared, the 1 L medium (except for CaCl_2_·2H_2_O and vitamins) was brought to boiling in order to remove O_2_, and it was later cooled down to room temperature under an oxygen-free N_2_ flow. Then CaCl_2_·2H_2_O and the vitamins were added, as well as Na_2_S (0.24 g) as reducing agent.

### 2.3 CO Fed Batch Reactor Set-Up

All experiments were carried out in 1 L fed batch reactors (Fisherbrand, FB-800-1100, Waltham, U.S.), with 10% enriched sludge in 200 ml medium. The fed batch reactors were sealed with a gas tight septum fitted with a pH probe (9,5 × 300 mm, VWR) in the middle. The pH probe was connected to a pH controller (Cole-Parmer 300, Cambridgeshire, United Kingdom) and the pH was adjusted using either 1 M NaOH or HCl solutions by two pumps (Verder International BV, Utrecht, the Netherlands). The fed batch reactors were agitated at 150 rpm by a shaker (Infors AG CH-4103, Bottmingen, Switzerland) at 33°C in a thermostatic chamber. 100% CO was supplied to the headspace of the reactor as the sole carbon and energy source with an initial gas pressure of 1.8 bar. When the gas pressure decreased below 1 bar, as a result of bacterial CO gas consumption (corresponding to one CO feeding), the reactor was flushed with fresh pure CO for about 5 min, until reaching again a gas pressure of 1.8 bar. Gas and liquid samples were taken once at each experimental time point and analyzed once.

### 2.4 Experimental Design

#### 2.4.1 Exogenous Acetic Acid, Ethanol and Butyric Acid Addition

**FIGURE 1:**
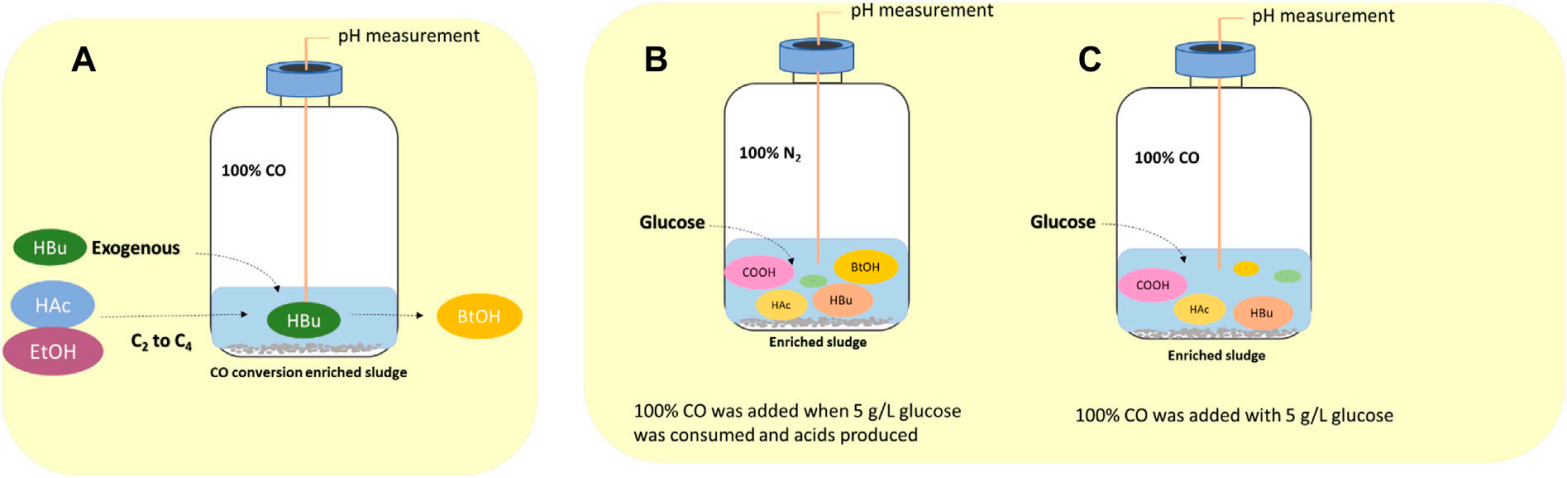
Schematic diagram of **(A)** influence of exogenous acetate, butyrate and ethanol on butanol production using CO as gaseous substrate, **(B)** endogenous butyric acid from glucose (Glucose + N_2_) and **(C)** co-fermentation of CO and glucose (Glucose + CO).

The effect of exogenous acetic acid, butyric acid and ethanol on the production of butanol by enriched sludge was evaluated in four 1 L fed batch reactors with 200 ml culture medium and inoculated with 10% enriched sludge (Section 2.2). The medium was supplied with acetic acid and ethanol (HAc + EtOH); acetic acid, ethanol and butyric acid (HAc + EtOH + HBu); or butyric acid (HBu), respectively, besides the control described below (Figure 1A).

In the HAc + EtOH experiment, acetate and ethanol addition was designed to stimulate the butyric acid production via the reverse β oxidation pathway and further induced butanol production using CO as reducing power. In the HAc + EtOH + HBu experiment, to demonstrate the enhancement of acetate and ethanol (reverse β oxidation pathway) on the butyric acid production, combined with exogenous butyric acid, which can possibly result in enhanced butanol production using CO as reducing power.

An initial pH of 5.9 was applied and the pH was adjusted to 5.9 at each CO addition. The final concentrations of acetic acid, butyric acid and ethanol were, respectively, 2.2, 3.2 and 6 g/L (Table 1). The acetic and butyric acid concentrations were chosen to obtain a molar relationship of ethanol to acid of around 3.5 that has been shown for enhanced butyric acid production. The control contained the same gas pressure and inoculum, but without acids and ethanol addition.

**TABLE 1:** The highest net acids and alcohol production, mole ratio of ethanol to acetic acid and butanol to butyric acid, butyric acid conversion and butanol yield of experiments on the influence of acetate, butyrate and ethanol on butanol production using CO as gaseous substrate.

No.	Gas	Exogenous acids and ethanol (g/L)			Molar ratio incubation medium		Highest net production (g/L)						Molar ratio products		Butanol yield (%)	Butyric acid conversion (%)
		Acetic acid	Butyric acid	Ethanol	Ethanol/Acetic acid	Ethanol/Butyric acid	Acetic acid	Butyric acid	Ethanol	Butanol	Propionic acid	Valeric acid	EtOH/HAc	BtOH/HBu		
I. Exogenous acetic acid, ethanol and butyric acid																
1	CO	2.2	0	6	3.55	—	11.2	1.1	**4.3**	1.6	1.3	0.2	1.79	3.84	—	—
2	CO	2.2	3.2	6	3.55	3.59	10.7	0.1	0.5	**2.6**	0.9	0.0	0.46	—	100.2	76.2
3	CO	0	3.2	0	—	—	6.9	0.1	**4.3**	1.9	1.3	0.1	0.33	—	67.0	53.7
4 Control	CO	0	0	0	—	—	8.3	0.6	2.7	0.2	0.4	0.0	3.45	—	—	—
II. Exogenous 7 and 14 g/L butyric acid																
5	CO	0	7	0	—	—	7.1	—	0.2	**2.3**	0.2	0	0.01	2.04	15.8	32.2
6	CO	0	14	0	—	—	3.9	—	0.1	**3.4**	0.1	0	0.05	1.16	58.0	67.5

CO was added at day 0, 11, 14, 15, 16 and 27 in the HAc + EtOH + HBu experiment; at 0, 11, 13, 15, 17, 20, 27 and 32 days in the HAc + EtOH experiment; at day 0, 17, 19, 20, 23, 25 and 30 in the HBu experiment; and at day 0, 20, 25, 27 and 35 in the control.

#### 2.4.2 Exogenous Butyric Acid Addition

Two 1 L CO fed batch reactors were used with 7 and 14 g/L butyric acid externally supplied at the same CO gas pressure and inoculum as described in Section 3.2. The pH value was maintained at 5.5–6.0 by 1 M HCl or 1 M NaOH. CO was added at day 0, 15 and 21 days in the 7 g/L HBu and at day 0, 20 and 25 days in the 14 g/L HBu incubation.

#### 2.4.3 Influence of Endogenous Butyric Acid From Glucose on Butanol Production Using CO as Gaseous Substrate

##### 2.4.3.1 Pre-Tests of Endogenous Butyric Acid From Glucose in CO Fed Batch Process

Endogenous butyric acid production from glucose was first tested in 125 ml serum bottles with 30 ml medium, and with initially 5 g/L glucose and N_2_ as the headspace (triplicates). When glucose had been totally consumed and butyric acid produced (glycolysis), the headspace was flushed with 100% CO for 5 min up to a final CO pressure of 1.8 bar. CO acts as reducing power to stimulate butanol production from endogenous butyric acid by the enriched *Clostridium* bacteria. CO was added at day 5, 9 and 11.

##### 2.4.3.2 Endogenous Butyric Acid From Glucose in CO Fed Reactor

The sequential conversion of glucose (glycolysis) and then CO (WLP) or the co-fermentation of glucose and CO by the enriched sludge were further investigated in two gas fed reactors (Figures 1B,C). pH was controlled at 5.5–6.2 by 1 M NaOH or 1 M HCl. Glucose was added initially to reach a concentration of 5 g/L and the headspace was flushed with either N_2_ or CO, i.e., Glucose + N_2_ and Glucose + CO. In the Glucose + N_2_ experiment, when glucose was totally consumed and butyric acid accumulated ((G + N)_1_), the headspace was flushed with CO for 5 min to reach a gas pressure of 1.8 bar at 180, 252, 360 and 432 h, respectively, to stimulate butanol accumulation ((G + N)_2_). In CO and glucose co-fermentation, CO was flushed at 0, 180, 276 and 432 h in the Glucose + CO experiment.

The microbial community of the four enriched sludge samples were analyzed: two samples from the Glucose + N_2_ experiment, when glucose was totally consumed and butyric acid accumulated ((G + N)_1_) and at the end of the incubation ((G + N)_2_) after CO was added as well as two samples from the Glucose + CO experiment, when glucose was totally consumed and butyric acid accumulated ((G + C)_1_), and at the end of the incubation ((G + C)_2_).

### 2.5 Analytical Methods

Acetic, propionic, and butyric acids, ethanol and butanol were determined on a high performance liquid chromatography (HPLC, HP1100, Agilent Co., Palo Alto, United States) equipped with a refractive index detector, using an Agilent Hi-Plex H Column (300 × 7.7 mm). A 5 mM H_2_SO_4_ solution was used as mobile phase at a flow rate of 0.80 ml/min, with a sample injection volume of 20 μl, and a column temperature of 45°C (Arslan et al., 2019). The cell concentration was determined with a spectrophotometer (Hitachi, Model U-200, Pacisa & Giralt, Madrid, Spain) at a wavelength of 600 nm (Arslan et al., 2019). pH was measured using a pH meter (Mettler Toledo, Zurich, Switzerland).

### 2.6 Calculations

The butyric acid (HBu) conversion efficiency and butanol (BtOH) yield were calculated according to Eqs 1, 2, respectively:

$$HBu\;conversion\;efficiency=\frac{C_{in}-C_{out}}{C_{in}},$$ (1)

where $C_{in}$ = the total carbon from exogenous butyric acid addition; $C_{out}$ = the carbon from the butyric acid at the end of the incubation after conversion to butanol.

$$BtOH\;yield=\frac{Real\;butanol\;production}{Theoretical\;butanol}\times 100\%$$ (2)

$$Theoretical\;butanol=\frac{C_{HBu}}{88}\times 74\times 4\times 1000,$$ (3)

where Real butanol production and Theoretical butanol were calculated in mmoL·L^−1^ C. Theoretical butanol was calculated from 100% conversion of the initial exogenous butyric acid in $\left(C_{HBu},\;g/L\right)$ in the HBu, HAc + EtOH and HAc + EtOH + HBu incubations. The molecular mass of butyric acid and butanol is, respectively, 88 g·moL^−1^ and 74 g·moL^−1^.

### 2.7 Microbial Analysis

DNA was extracted using a DNeasy^®^ PowerSoil Kit (QIAGEN, Germany) following the manufacturer’s protocol. 10 ml sludge was used for DNA extraction at the end of the incubations. The extracted DNA was quantified and its quality was checked with a Nanodrop 2000c Spectrophotometer (Thermo Scientific, United States). The extracted DNA was analyzed by Metagenomics -Seq (Illumina PE150, Q30 ≥ 80%) (Novogene, United Kingdom). The procedure has been described in He et al. (2021a).

## 3 Results

### 3.1 Ethanol and Butanol Production With Exogenous Acetic Acid, Ethanol and Butyric Acid Using CO as Gaseous Substrate

#### 3.1.1 Exogenous Acetic Acid and Ethanol Supply

**FIGURE 2:**
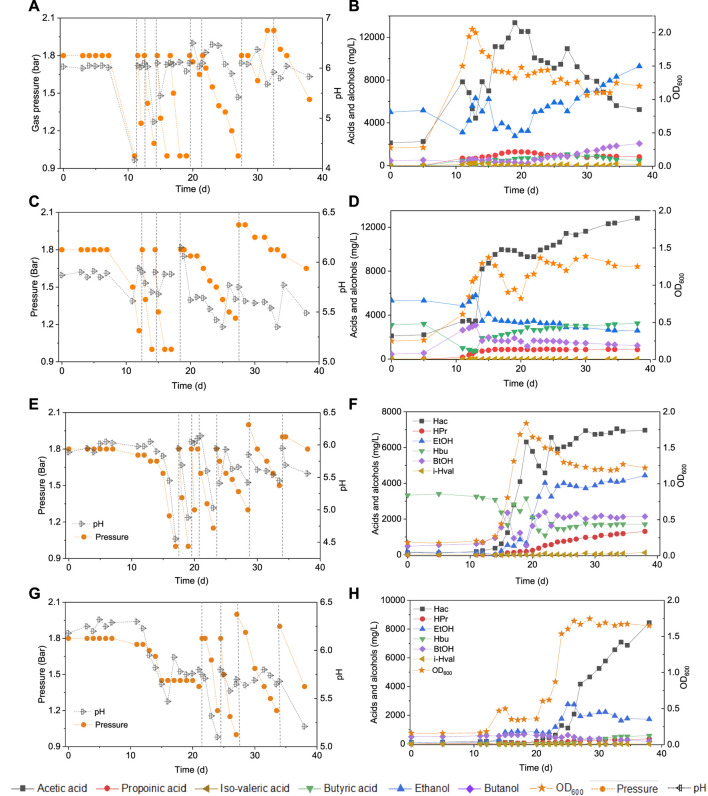
Production of acids and alcohols, cells concentration and change of gas pressure in semi-gas feeding reactors by enriched sludge from 6^th^ transfer using CO as the carbon source. **(A)** and **(B)** exogenous acetic acid and ethanol; **(C)** and **(D)** exogenous acetic acid, ethanol and butyric acid; **(E)** and **(F)** exogenous butyric acid; **(G)** and **(H)** control without exogenous acids or ethanol. The dashed lines represent CO addition and pH regulation.

In the HAc + EtOH experiment, butanol production was not observed till day 20 (Figures 2A,B). Thereafter, interestingly, butanol production increased and reached a final concentration of 2.1 g/L at the end of the incubation, during which the pH ranged between 5.4 and 6.5 (Figures 2A,B). However, acetic acid was produced first and increased from 2.2 g/L to 7.8 g/L and simultaneously the ethanol concentration decreased from 5.0 g/L to 3.1 g/L on day 11. The pH dropped to 4.07 due to the accumulation of acetic acid and the biomass reached an OD_600_ of 1.5 (Figures 2A,B). Thereafter, acetic acid accumulated to its maximum concentration of 12.5 g/L (Figure 2B), while the ethanol concentration quickly increased to 6.3 g/L on day 15 to finally decrease again to as low as 3.5 g/L on day 20. At the seventh CO addition (day 20–25), ethanol increased again to 5.9 g/L at day 25 (Figure 2B). At the last CO addition, the CO gas pressure was increased to 2.0 bar, ethanol increased again and reached its highest concentration of 9.3 g/L at pH 5.8–6.4. The highest net production of acetic acid, ethanol, butyric acid and butanol reached, respectively, 11.2, 4.3, 1.1 and 1.6 g/L (Table 1). Other acids such as propionic acid with the highest concentration of 1.3 g/L and a small amount of valeric acid (0.2 g/L) were obtained during the fermentation (Table 1).

#### 3.1.2 Exogenous Acetic Acid, Ethanol and Butyric Acid Supply

In the HAc + EtOH + HBu experiment, the butanol concentration increased to 2.8 g/L along with the butyric acid concentration dropping from 3.1 to 1.0 g/L, with the pH ranging from 5.9 to 5.6, at the first CO addition (Figures 2C,D). Ethanol slightly decreased while acetic acid increased after a few days of adaption, which exhibits a similar trend as in the HAc + EtOH experiment (Figures 2B,D). On the second CO addition (day 11–14), butanol increased to 3.1 g/L at day 13, when the gas pressure dropped to 1.4 bar (Figures 2C,D). During the gas pressure decrease from 1.4 to 1 bar, the concentrations of both butanol and ethanol decreased, respectively, from 3.1 to 1.7 g/L and 8 to 3.5 g/L on day 13–14. Conversely, the butyric acid concentration increased from 0.7 to 1.9 g/L and acetic acid accumulated quickly to 8.2 g/L (Figures 2C,D). Thereafter, acetic acid reached 10.0 g/L at day 16 while butyric acid increased continuously until the end of the incubation (Figures 2C,D). After 38 days incubation, the highest net acetic acid concentration of 10.7 g/L was obtained; while the highest net production of ethanol, butyric acid, and butanol reached, respectively, 0.5, 0.1, and 2.6 g/L (Table 1).

#### 3.1.3 Exogenous Butyric Acid Supply

In the exogenous HBu experiment, the butanol concentration increased to 2.4 g/L along with the exogenous butyric acid concentration dropping from 3.2 to 1.7 g/L, at pH 5.9, on day 16 (Figure 2F). However, later on, the butanol concentration decreased from 2.4 to 1.0 g/L, at day 17, with a pH drop from 5.45 to 4.45, and the gas pressure decreasing from 1.25 to 1.0 bar (Figures 2E,F). Acetic acid increased to 2.8 g/L, while the pH value decreased to 4.55 and ethanol slightly increased to 0.5 g/L. At the second CO addition (17–19 days), butanol dropped again to its lowest concentration of 0.5 g/L, while butyric acid increased to 3.2 g/L at day 19 (Figure 2F). The acetic acid concentration increased to 6.3 g/L, along with the pH drop to 4.88 (Figure 2E). At the third and fourth CO addition (19–23 days), ethanol and butanol increased again accompanied by the decrease of acetic acid and butyric acid concentrations, reaching 6.9 g/L acetic acid, 4.4 g/L ethanol, 1.7 g/L butyric acid and 2.1 g/L butanol at day 38 (Figure 2F). The propionic acid concentration increased after day 20 and reached 1.3 g/L at day 38 (Figure 2F).

In the control, five CO additions were amended (Figure 2G). Acetic acid and ethanol were mainly produced, with the highest concentrations of, respectively, 8.2 and 2.8 g/L (Figure 2H). Biomass growth was also observed (Figure 2H). Both butyric acid and butanol were insignificantly produced, with only 0.6 g/L butyric acid and 0.2 g/L butanol production at day 38 (Figure 2H).

### 3.2 Effect of Exogenous Butyric Acid Supply on Butanol Production

**FIGURE 3:**
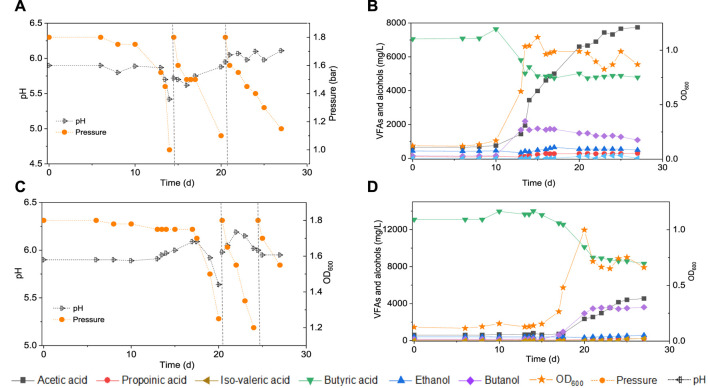
Production of acids and alcohols, cells concentration and change of gas pressure in semi-gas feeding reactors by enriched sludge from 6^th^ transfer (He et al., 2021a) using CO as the carbon source. **(A)** and **(B)** exogenous 7 g/L butyric acid; **(C)** and **(D)** exogenous 14 g/L butyric acid. The dashed lines represent CO addition and pH regulation.

Upon 7 g/L butyric acid addition, the butanol concentration increased to 2.2 g/L at day 13 while butyric acid decreased from 7.0 to 5.0 g/L (Figure 3B). Simultaneously, acetic acid increased to 1.9 g/L at day 13 and 4.0 g/L at day 15 and biomass growth entered into the log phase and with the OD_600_ reaching to the highest value of 1.1 (Figure 3B). Then, the butanol concentration slightly decreased to 1.1 g/L while acetic acid increased to its highest concentration of 7.8 g/L at day 38 (Figure 3B). A small amount of hexanoic acid was observed with the highest concentration of 0.2 g/L at day 25 and propionic acid increased to 0.3 g/L at day 38 (Figure 3B).

In a second assay, with 14 g/L exogenous butyric acid supply, butanol increased to 2.9 g/L at day 20 along with the amount butyric acid dropping (Figure 3D). Butanol kept increasing to 3.5 g/L at day 25 and finally reached 3.6 g/L at the third CO addition, at day 27 (Figure 3D). The acetic acid concentration increased to 2.3 g/L at day 20, with the cell concentration reaching its highest absorbance of 1.000 (Figure 3D). At the end of the incubation (day 38), acetic acid had increased to its highest concentration of 4.5 g/L, while the cell concentration (OD_600_) decreased to 0.661 (Figure 3D). The butanol production pattern was similar to the exogenous supply of 7 g/L butyric acid, except that the butanol accumulation was twice as high. The butanol production slowed down and even stopped after the first CO addition, but acetic acid production and biomass growth continued.

Figure 2 and Table 1 thus show that the butanol production was significantly enhanced in the presence of exogenous butyric acid. The amount of accumulated butanol increased up to 1.2 and 1.8 fold, respectively, with 7 and 14 g/L HBu, compared to 3.2 g/L HBu. Although the butanol yield reached its highest value of 67.0% with 3.2 g/L HBu, it decreased to, respectively, 15.8 and 58.0% with 7 and 14 g/L HBu (Table 1). The butyric acid conversion efficiency reached its highest value of 67.5% with 14 g/L HBu, correspondingly 1.3 and 2.1 fold higher than, respectively, than 7 and 3.2 g/L HBu (32.2 and 53.7%, respectively) (Table 1). However, in the presence of HAc + EtOH + HBu, the butanol yield and butyric acid conversion efficiency reached, respectively, 100 and 76.2% which are both higher than with HBu (Table 1).

### 3.3 Fermentation With Endogenous Butyric Acid From Glucose

#### 3.3.1 Batch Tests

**FIGURE 4:**
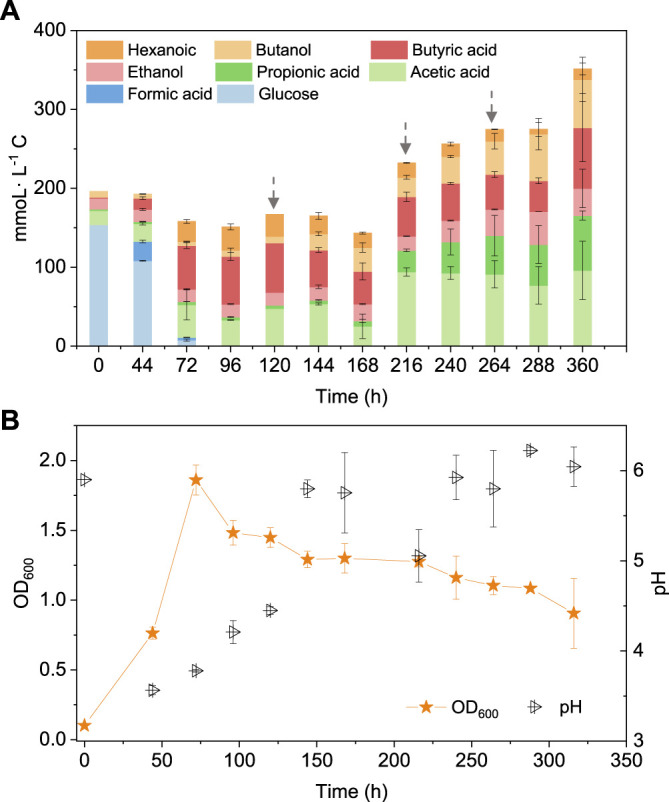
**(A)** Production of acids and alcohols and **(B)** cell concentration and pH by enriched sludge using 5 g/L glucose as the substrate in batch tests. The arrows in **(A)** represent 1.1 bar CO addition at 120, 216 and 264 h. pH was adjusted to 5.9 on a daily basis.

To better understand the carbon flow, the liquid products are shown in mmoL L^−1^ C in a bar chart (Figure 4). Initially 154 mmoL·L^−1^ C was added in the form of glucose (5 g/L) with a small amount of additional carbon carried over from the inoculum (Figure 4). After 44 h, the accumulation of 24 mmoL·L^−1^ C formic acid was detected, which was then quickly converted to acetic acid, butyric acid and propionic acid with, respectively, 23.4, 54.0 and 2.1 mmoL·L^−1^ C at 72 h, when glucose was totally consumed (Figure 4A). Butyric acid reached a concentration of 59.2 mmoL·L^−1^ C at 96 h and remained relatively stable thereafter (Figure 4A). Therefore, CO was added at 120 h when all the original substrate (glucose) was exhausted. Then, butanol was produced and increased from 8 mmoL·L^−1^ C at 120 h to, respectively, 21 and 30 mmoL·L^−1^ C at 144 and 168 h. To stimulate butanol production, CO was again added at 168, 216 and 264 h (Figure 4A). Correspondingly, the butanol concentration continuously increased to 61 mmoL·L^−1^ C, representing a net production of 53.6 mmoL·L^−1^ C, at the end of the incubation (Figure 4A; Table 2). Ethanol production was also observed with the CO addition, reaching 34 mmoL·L^−1^ C at the end of the incubation (Figure 4A).

**TABLE 2:** The highest net production of acids and alcohol, mole ratio of butanol to butyric acid and butanol yield.

Experimental conditions		The highest net production (mmoL·L^−1^ C)							BtOH/HBu	HBu conversion efficiency (%)	Butanol yield (%)
		Formic acid	Acetic acid	Butyric acid	Ethanol	Butanol	Hexanoic acid	Propionic acid			
pH control	5 g/L Glucose + N_2_ then CO	95.4	17.5	42.9	6.6	26.6	33.0	4.7	1.50	58.6	62.1
	5 g/L Glucose + CO	70.3	60.4	50.3	1.0	11.1	0	34.2	0.35	45.3	22.1
Batch tests	5 g/L Glucose + N_2_ then CO	24.5	77.9	75.7	82.0	53.6	29.9	67.7	0.71	—	—

#### 3.3.2 Endogenous Butyric Acid Production From Glucose and its Conversion to Butanol in a Gas Fed Reactor With pH Control

**FIGURE 5:**
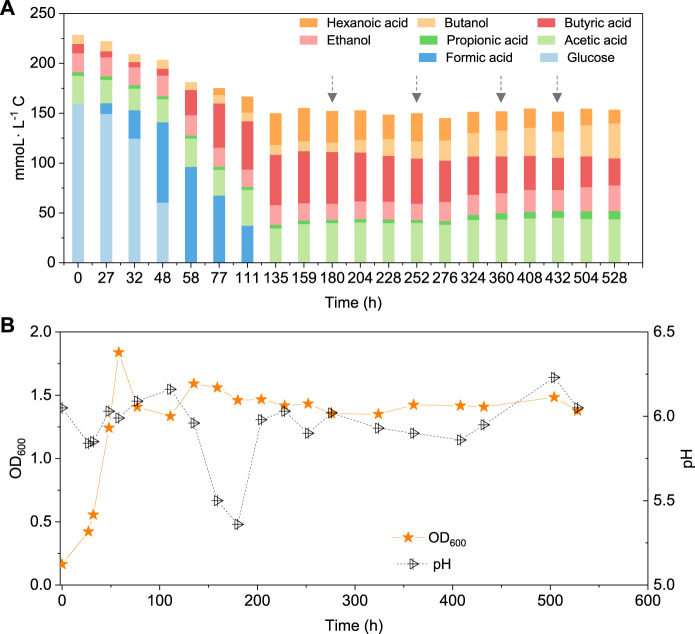
**(A)** Production of acids and alcohols and **(B)** cell concentration and pH by enriched sludge using 5 g/L glucose as the substrate in a gas fed reactor (Glucose + N_2_). The arrows represent 1.1 bar CO addition at 180, 252, 360 and 432 h. pH was controlled at 5.5–6.2.

When using 5 g/L glucose as the substrate, glucose was totally consumed after 58 h, with a production of 95.4 mmoL·L^−1^ C formic acid, 0.7 mmoL·L^−1^ C acetic acid and 16.1 mmoL·L^−1^ C butyric acid (Figure 5A). Formic acid was then subsequently consumed and converted to 6.9 mmoL·L^−1^ C acetic acid, 41.0 mmoL·L^−1^ C butyric acid and 31.2 mmoL·L^−1^ C hexanoic acid at 135 h (Figure 5A). The pH value decreased to 5.5 at 180 h, due to the accumulation of acids, and it was then adjusted back to 6.0 (Figure 5B). After CO addition at 180 h, butanol production was triggered and increased from 1.1 mmoL·L^−1^ C at 180 h to 4.2 mmoL·L^−1^ C, at 204 h, and later 8.7 mmoL·L^−1^ C, at 252 h (Figure 5A). CO was then supplied again later, at 252, 360 and 432 h to potentially stimulate butanol production. Consequently, the net butanol production increased to 17.4 mmoL·L^−1^ C at 360 h, and finally 26.6 mmoL·L^−1^ C at the end of the incubation and the butanol yield reached 62.1% (Figure 5A; Table 2). The remaining butyric acid concentration was 17.8 mmoL·L^−1^ C at the end of the incubation, and the net butyric acid consumption was 24.7 mmoL·L^−1^ C, calculated from the difference with 42.5 mmoL·L^−1^ C at 180 h, when CO was first added. This almost equals the net butanol production of 25.5 mmoL·L^−1^ C at the end of the incubation (Figure 5A).

#### 3.3.3 Co-Fermentation of Glucose and CO in a Gas Fed Reactor With pH Control

**FIGURE 6:**
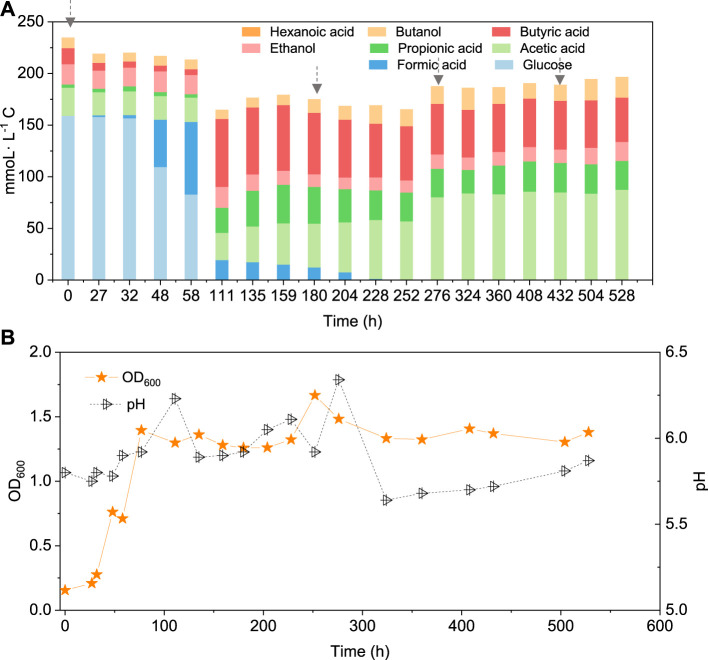
**(A)** Production of acids and alcohols and **(B)** cell concentration and pH by enriched sludge from 6^th^ transfer using 5 g/L glucose and CO as substrate in a gas fed reactor with intermittent CO gas feeding (Glucose + CO). The arrows in a) represent 1.1 bar CO addition at 0, 180, 276 and 432 h, respectively. pH was controlled at 5.5–6.2.

In the co-fermentation of CO and glucose (5 g/L), half the amount of glucose was consumed after 58 h, along with the production of 70 mmoL·L^−1^ C formic acid, as the major end product (Figure 6A). Later, glucose was totally consumed, at 111 h, and 19.5 mmoL·L^−1^ C formic acid, 21.2 mmoL·L^−1^ C propionic acid and 50.3 mmoL·L^−1^ C butyric acid were produced (Figure 6A). CO was added at 180, 276 and 432 h to enhance butanol production. The butanol concentration did indeed increase to 6.8 mmoL·L^−1^ C, after 276 h, 5.3 mmoL·L^−1^ C, after 432 h, and 9.7 mmoL·L^−1^ C at the end of the incubation (Figure 6A). Both acetic acid and propionic acid reached as high as 60.4 and 34.2 mmoL·L^−1^ C, respectively, at the end of the incubation. Besides, the butanol yield reached 22.1% at the end of the incubation (Figure 6A).

### 3.4 Microbial Analysis

**FIGURE 7:**
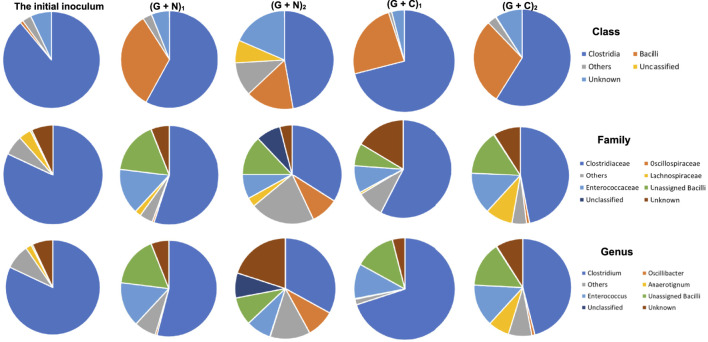
The microbial community analysis of the initial inoculum, after glucose fermentation and CO and glucose co-fermentation from 6^th^ transfer. “(G + N)_1_” represents when glucose was totally consumed using glucose as substrate and “(G + N)_2_” represents at the end of the incubation after CO was added. “(G + C)_1_” represents when glucose was totally consumed and “(G + C)_2_” represents at the end of the glucose and CO co-fermentation incubation.

When supplying glucose + N_2_, the relative abundance of the *Clostridia* and *Bacilli* class in the initial inoculum was, respectively, 82 and 1%, and reached, respectively, 58 and 33% when glucose was totally consumed ((G + N)_1_, glycolysis). Then, both decreased to, respectively, 51 and 17% and unassigned bacteria occupied 8% at the end of the incubation [after replacing the headspace with CO, (G + N)_2_] (Figure 7). In the *Clostridia* class, the *Clostridium* genus decreased from 54 to 33%, while *Oscillibacter* genus increased from 0.6 to 9% at the end of the incubation. In the *Bacilli* class, the *Enterococcus* genus decreased from 15 to 8% (Figure 7).

At the *Clostridium* genus level, *Clostridium butyricum* increased from 0.003% in the initial inoculum to 16% when glucose was totally consumed, while it decreased to 6% at the end of the incubation, after replacing the headspace with CO (Table 3). *C. carboxidivorans* was enriched from 0.3% of the whole *Clostridium* genus in the inoculum to, respectively, 6% when glucose was totally consumed and 4% at the end of the incubation (Table 3). The relative abundance of other known *Clostridium* species with relative abundance higher than 2% increased much at the end of the incubation compared to when glucose was totally consumed (Table 2). For instance, the relative abundance of *Clostridium* sp. W14A increased from 0.04 to 3%, *Clostridium ragsdalei* from 0.07 to 4%, *C. ljungdahlii* from 0.1 to 4%, *C. autoethanogenum* from 0.03 to 2%, and *Clostridium coskatii* from 0.07 to 2% (Table 3).

**TABLE 3:** Relative abundance of *Clostridium* spp. at species level in Glucose + CO and Glucose + N_2_ by enriched sludge from 6^th^ transfer.

		Initial inoculum	Glucose		Glucose + CO	
			(G + N)_1_	(G + N)_2_	(G + C)_1_	(G + C)_2_
Clostridium genus	Of bacteria	88	58	42	72	51
	Of root	82	54	33	70	46
Species name		*Clostridium* (Genus level) %				
*C. butyricum*		0.003	16	6	17	2
*C. carboxidivorans*		0.3	6	4	0.1	6
*Clostridium* sp. C8		0.006	3	3	0.5	0.3
*Clostridium* sp. IBUN125C		0.00001	2	0.8	4	0.5
*Clostridium cadaveris*		0.1	3	0.9	0.008	0.4
*Clostridium* sp. W14A		0.4	0.04	3	0.08	0.8
*C. ragsdalei*		4	0.07	4	0.006	3
*C. ljungdahlii*		8	0.1	4	0.006	6
*C. autoethanogenum*		6	0.03	2	0.002	4
*C. coskatii*		4	0.07	2	0.003	3
*C. kluyveri*		0.3	0.02	0.3	0.004	0.2
Other identified *Clostridium* spp.		3.9	10.7	12.0	3.3	8.8
Unassigned *Clostridium* spp.		73	59	58	75	65

In CO and glucose co-fermentation (Glucose + CO), the enriched sludge was dominated by both *Clostridia* and *Bacilli* classes with relative abundance of 71 and 24%, respectively, when glucose was totally consumed ((G + C)_1_). Then, it changed to, respectively, 59 and 29% at the end of the incubation ((G + C)_2_) (Figure 7). In the *Clostridia* class, although the *Clostridiaceae* family still occupied as high as 47% at the end of the incubation, an increase of other families was also observed, such as the *Lachnospiraceae* family increasing from 0.8%, in (G + C)_1_–9% and the *Oscillospriraceae* family increasing from 0.08 to 1% in (G + C)_2_ (Figure 7).

At the *Clostridium* genus level, the relative abundance of *C. butyricum* reached 17% in (G + C)_1_ but it then decreased to 2% in (G + C)_2_ (Table 3). *C. carboxidivorans* significantly increased to 6% in (G + C)_2_ compared to 0.1% in (G + C)_1_ (Table 3). The relative abundance of other species such as *Clostridium* sp*.* W14A increased from 0.08 to 0.8%, *C. ragsdalei* from 0.006 to 3%, *C. ljungdahlii* from 0.006 to 6%, *C. autoethanogenum* from 0.02 to 4%, and *C. coskatii* from 0.003 to 3% (Table 3).

**FIGURE 8:**
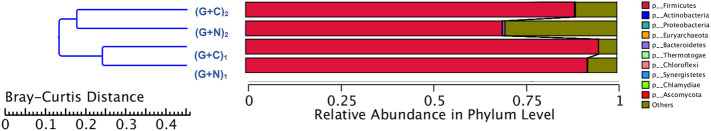
Clustering tree based on Bray-Curtis distance of the microbial community after G + N and G + C fermentation by enriched sludge from 6^th^ transfer. “(G + N)_1_” represents when glucose totally consumed using glucose as substrate and “(G + N)_2_” represents at the end of the incubation after CO was added. “(G + C)_1_” represents when glucose totally consumed and “(G + C)_2_” represents at the end of the incubation in glucose and CO co-fermentation.

**FIGURE 9:**
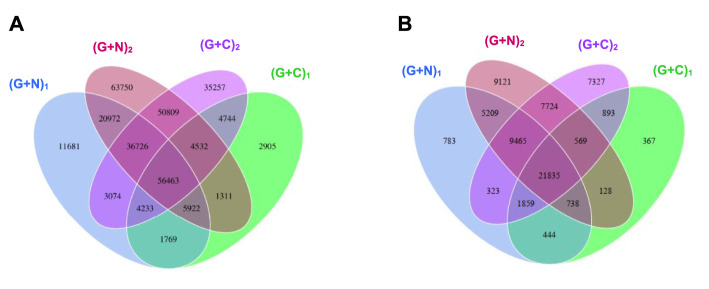
Venn diagrams of gene number of **(A)** the total genes and **(B)** the genes in *Clostridium* genus level. The overlap parts represent the number of common genes between/among samples (groups); the other parts represent the number of special genes of samples (groups). “(G + N)_1_” represents when glucose was totally consumed using glucose as substrate and “(G + N)_2_” represents at the end of the incubation after CO was added. “(G + C)_1_” represents when glucose was totally consumed and “(G + C)_2_” represents at the end of the incubation in glucose and CO co-fermentation.

Figure 8 shows the clustering tree based on Bray-Curtis dissimilarity and the relative abundance at phylum level among (G + N)_1_, (G + N)_2_ (G + C)_1_ and (G + C)_2_. (G + N)_1_ had high dissimilarity with (G + C)_1_, while (G + N)_2_ had high similarity with (G + C)_2_, but these four enriched sludges still exhibited close distances (Figure 8). The common and special genes among the transfers is shown in the Venn figure (Figure 9). In the core common genome of 56,463 genes, special genes increased at the end of the incubation. For instance, 2,905 special genes appeared in (G + C)_1_ while they increased significantly, to 35,257, in (G + C)_2_ (Figure 9A). Considering the high abundance of the *Clostridium* genus, a Venn figure for gene analysis at *Clostridium* genus level was done. Special genes that increased at the end of the incubation also showed a similar trend at *Clostridium* genus level (Figure 9B).

## 4 Discussion

### 4.1 Exogenous Butyric Acid Enhanced Butanol Production Using CO as Reducing Power

This study showed that butanol production was enhanced in the presence of exogenous butyric acid using CO as reducing power by the enriched sludge. Butanol production was increased along with increasing exogenous butyric acid concentrations (Table 1), which can be attributed to the increased extent of acid to alcohol conversion, according to Eq. 5:

$$C_{4}H_{7}O_{2}^{-}+H^{+}+CO\to C_{4}H_{10}O+{\text{CO}}_{2}\;∆G^{0}=-36.5\;\text{kJ }{\text{mol}}^{-1}$$ (5)

The highest butanol production of 3.4 g/L, with the exogenous supply of 14 g/L butyric acid is, to the best of our knowledge, the highest reported butanol concentration using CO as the gaseous substrate, except for one recent study reporting 6.8 g/L butanol production with a highly enriched mixed culture (He et al., 2022). Another previous research reported a butanol production of 2.66 g/L, together with 5.55 g/L ethanol from CO fermentation, with *C. carboxidivorans* at pH 5.75 (Fernández-Naveira et al., 2016). Butanol production occurred along with exogenous butyric acid consumption and kept increasing when cell growth entered the log phase (Figure 2). Acetic acid accumulation occurred rather later and accumulated fast after butanol reached a high concentration (Figure 2). This indicates the preference for butanol production in the presence of butyric acid and a 1.8 bar CO gas pressure, compared to acetic acid production. Furthermore, butanol production increased very quickly and reached close to the highest concentration at the end of the first CO addition (Figure 2 and Figure 3). However, it did not increase, instead, even slightly decreased after the first CO addition, which occurred almost in every experiment supplied with exogenous butyric acid (Figure 2). During the fermentation process, CO can be used as both carbon source for acetic acid production and reducing power to reduce butyric acid to butanol by the enriched sludge. When exogenous butyric acid was present in the medium at the start of the fermentation, acetogens took first advantage of the existing butyric acid, using CO as reducing power and reduced butyric acid to butanol. Along with the biomass growth adaption, acetogens obtained energy from acetic acid production (ATP releasing process), thus CO acted more as the carbon source inducing increased acetic acid production than reducing power for butanol production from butyric acid (Mock et al., 2015; Zhu et al., 2020). On the other hand, the fed batch pressurized CO feeding in sealed reactors may cause more CO_2_ solubilization in the liquid phase and cause a pH decrease, especially in uncontrolled pH processes. When the pH decreases below the pKa of the volatile fatty acids (4.75 for acetic acid, 4.8 for butyric acid), the undissociated acid can enter the cells and may thus inhibit biomass activity and growth (He et al., 2020b). Thus, continuous gas fed reactors for butanol fermentation are preferably operated at controlled pH.

Butanol production occurred at pH 5.9 and with a CO gas pressure as high as 1.8 bar; but when the pH was lower than 5.5, butanol production was inhibited (Figure 2). This pH range for alcohol production is in agreement with previous studies, in which the pH ranged from 5.7 to 6.4 for selective butanol production by the same enriched sludge as used in this study (He et al., 2022). Instead, the pH range is different from other previous reports, in which solventogenesis was stimulated at lower pH, varying between 4.5–5.5 (Ganigué et al., 2016; Chakraborty et al., 2019). The wide pH range for butanol production might be due to the mixed *Clostridium* spp. present in the inoculum used in this study. For instance, the relative abundance of *C. autoethanogenum* and *C. ljungdahlii* occupied, respectively, 6 and 8%, which are known to convert CO to acetic acid and ethanol via the WLP while butanol-producing *C. carboxidivorans* abundance was less than 1% (He et al., 2021a). However, the relative abundance of unidentified *Clostridium* species occupied as high as 73% of the *Clostridium* genus (He et al., 2021a).

The mole ratio of butanol production and butyric acid consumption with exogenous 3.2 g/L HBu is close to 1 during the fermentation process (Figures 2D,F), which agrees with the theoretical conversion ratio (Eq. 5), demonstrating that the conversion occurs according to the stoichiometry given in Eq. 5. The mole ratio of butanol to butyric acid reached 2.04 with the exogenous supply of 7 g/L butyric acid, which is higher than 1.16 with exogenous supply of 14 g/L butyric acid (Table 1).

In addition, the adaption stage for cell growth and the end production was also shorter compared to the control, which all indicated that exogenous butyric acid can somewhat relief CO toxicity for acetogens and thus allow these acetogens to use the existing butyric acid to produce butanol using CO as reducing power. Besides, with higher exogenous butyric acid concentrations, acetic acid production was somewhat inhibited and, simultaneously, longer adaption times were observed for butanol production. For instance, the highest acetic acid production with exogenous supply of 3.2 and 7.0 g/L HBu is both 1.8 fold higher (7.1 and 6.9 g/L) than with 14 g/L HBu (3.9 g/L) (Figures 3B,D; Table 1).

### 4.2 The Presence of Acetate and Ethanol Favored Butanol Production From Butyric Acid Using CO as Reducing Power

This study showed that the presence of acetic acid and ethanol played a positive role in accelerating butanol conversion from exogenous butyric acid. In the HAc + EtOH + HBu experiment, the butanol yield and butyric acid conversion efficiency reached 100 and 76.2%, respectively, which were both higher than in the HBu experiment (Table 1). However, seldom studies have investigated the role of exogenous acetate and ethanol on butanol production using CO as gaseous substrate, except a few studies on exogenous butyric acid production by pure stains (Luo et al., 2015; Xu et al., 2020). Isom et al. (2015) conducting conversion efficiency studies of butyric acid to butanol, reaching 100% with *C. ragsdalei*. In that study, the reduction was independent of growth in an optimized medium with headspace CO gas exchange every 48 h and with a gas pressure of 207 kPa. Perez et al. (2013) investigated exogenous 15 mM (1.32 g/L) butyric acid addition on butanol production by *C. ljungdahlii* ERI-2 using syngas, CO/H_2_/CO_2_ (60/35/5, v/v/v), and reached 13.3 mM (0.99 g/L) *n*-butanol and 91% of the theoretical yield from butyric acid. Liu et al. (2014b) reported 74.7% butyric acid conversion to *n*-butanol by a mixed culture of *Alkalibaculum bacchi* strain CP15 and *Clostridium propionicum* and obtained 0.8 g/L butanol with 1.5 g/L exogenous butyric acid, using syngas (CO/CO_2_/H_2_, 40/30/30, v/v) as gaseous substrate.

A decrease of both the butanol and ethanol concentrations was observed during the fermentation process when the gas pressure dropped below 1.3 bar (Figure 2). This was induced by the production and accumulation of CO_2_ during CO consumption (Eqs 1, 2). More CO_2_ dissolved into the medium than CO due to its higher solubility, which further caused a decrease in gas pressure. Butanol and ethanol oxidation occurred in the presence of CO_2_ according to our previous tests using the same inoculum as used in this study (He et al., 2022). Besides, the reverse reaction of alcohols to acids has sometimes also been observed (Arslan et al., 2019). It seems that ethanol and butanol production, or reversely, oxidation, are regulated by, respectively, the CO and CO_2_ concentrations. Indeed, when CO was added at the beginning of each CO addition (1.8 bar) and CO_2_ was removed at the same time, alcohol production was favored (Figure 2). The increased CO pressure may play a positive role inducing solventogenesis. For instance, increasing the CO pressure to 2 bar in the last two CO additions of the exogenous HAc + EtOH + HBu experiment, induced a higher ethanol production (Figure 2B).

It was noted that a longer adaption time was required in the control bottle compared to the bottles with acids and ethanol addition. This might be because the added acetic acid, ethanol and butyric acid can act as carbon sources and thus somewhat relief the possible toxicity caused by CO at a pressure as high as 1.8 bar. The mole ratios of ethanol production to acetic acid consumption are all close to 1, which is in agreement with the theoretical mole ratio of the WLP (Figure 3A, Eq. 6):

$$C_{2}H_{3}O_{2}^{-}+H^{+}+CO\to C_{2}H_{6}O+{\text{CO}}_{2}\;∆G^{0}=-9.0\;\text{kJ }{\text{moL}}^{-1}$$ (6)

### 4.3 CO Triggered Butanol Production From Endogenous Butyric Acid Produced From Glucose With no Significant Microbial Community Change

This study showed that endogenous butyric acid was successfully produced from glucose and CO addition triggered butanol production from the existing endogenous butyric acid (Figure 4) and the mole ratio of butanol production to butyric acid consumption was close to 1 (Eq. 5). Publications on exogenous acids addition for enhanced alcohol production are rather scarce. The highest butanol production reached 0.5 g/L with *Alkalibaculum bacchi* strain CP15, and it was further enhanced to 0.8 g/L with *C. propionicum* using syngas CO/CO_2_/H_2_ (40/30/30, v/v/v) as the substrate (Liu et al., 2014b). This study promoted “syngas-aided” alcohol production, i.e., enhancing butanol and longer chain alcohol production by taking advantage of the CO reducing power in triggering alcohol production with exogenous acids or acids produced from waste organic compounds.

The microbial community does not show large differences in (G + N)_1_ and (G + N)_2_ in terms of *Clostridia* and *Bacilli* classes. The *Oscillibacter* genus, belonging to the *Clostridia* class, was enriched at the end of the incubation ((G + N)_2_) and has been observed to be involved in acidogenesis during dark fermentation (Goud et al., 2017). It was surprising that *C. carboxidivorans,* known as one of the autotrophic CO-converting acetogens, was enriched in (G + N)_1_, while its abundance decreased compared to the inoculum (Table 3). *C. carboxidivorans* can grow with glucose to produce formic, acetic, butyric and hexanoic acids at a relatively high pH of 6.2, while a low pH did not favour alcohol production (Fernández-Naveira et al., 2017b). *C. butyricum* can produce acetate and butyrate using glucose as the substrate and has also been shown to produce H_2_ (Crabbendam et al., 1985; Jiang et al., 2016). The heterotrophic *C. butyricum* species was enriched when glucose was totally consumed. However, thereafter, when CO was used as the substrate, the relative abundance of *C. butyricum* decreased, while observing the enrichment of autotrophic acetogens such as *C. ljungdahlii, C. autoethanogenum, Clostridium* sp. W14A and *C. ragsdalei*, which increased again using CO as the substrate (Table 3).

### 4.4 Glucose and CO Co-Fermentation Enhanced Butyric Acid but not Butanol Production

Interestingly, butanol was insignificantly produced in glucose and CO co-fermentation, although butyric acid accumulated and reached 50.3 mmoL·L^−1^ C, which is even slightly higher than the highest butyric acid concentration (42.9 mmoL·L^−1^ C) reached with only glucose (Figure 6; Table 2). In other words, different ways of adding CO may lead to different butanol concentrations: observing almost 2.4 fold more butanol (26.6 compared to 11.1 mmoL·L^−1^ C) when adding CO after endogenous butyric acid than initially adding CO (CO and glucose co-fermentation) at a pH controlled at 5.8–6.0. Co-fermentations of CO or syngas with glucose has only been reported with a few pure cultures as mentioned above, e.g., *C. carboxidivorans* (Fernández-Naveira et al., 2017b). It was supposed that in Glucose + N_2_, when the sole carbon source (glucose) was consumed along with acids produced, another inorganic carbon source (CO) addition would stimulate autotrophic acetogens to convert butyric acid to butanol due to its reductant role (Figure 5). This is similar with the exogenous butyric acid experiments: butyric acid was first consumed and converted to butanol using CO as reducing power by the enriched sludge, earlier than acetic acid production from CO (Figures 2B,C, Figure 3). In G + C, CO acted more as the carbon source instead of reducing power, resulting in higher acetic acid production (Figure 6A). Therefore, further research should focus the enhancement of butanol production with endogenous butyric acid produced from cheaper organic compounds than glucose in continuous gas fed bioreactors with controlled pH.

The presence of CO decreased the glucose consumption rate with only half of the initial amount of glucose consumed after 58 h, when glucose was totally consumed in Glucose + N_2_ (Figure 6A). Secondly, both acetic acid and propionic acid concentrations were much higher compared to Glucose + N_2_. Hexanoic acid production was inhibited in the Glucose + CO incubation, while its production reached as high as 33 mmoL·L^−1^ C in Glucose + N_2_. The highest hexanoic acid production of 31.2 mmoL·L^−1^ C in Glucose + N_2_ occupied 18.9% of the initial carbon of 165.9 mmoL·L^−1^ C from 5 g/L glucose (Table 2). Although butanol was not efficiently produced, the glucose and CO co-fermentation might provide another possible way for short chain fatty acids production by the enriched sludge with a highly abundant *Clostridium* spp. population.

The microbial community in the Glucose + CO incubation did not show large changes compared to Glucose + N_2_ (Figure 8). In the Glucose + CO incubation, the abundance of the *Clostridium* genus increased, i.e., the relative abundance of *Clostridium* genus with Glucose + CO (70%) was higher than with Glucose + N_2_ (54%) (Figure 8). The relative abundance at the genus level was distributed as follows (Figure 7): *Clostridium* 33%, *Oscillibacter* 8% and *Anaerotignum* 0.1% in (G + C)_1_ while *Clostridium* genus 46%, *Oscillibacter* 1% and *Anaerotignum* 7% in (G + N)_1_. The *Bacilli* class occupied 29% in G (G + C)_2_ and 17% in (G + N)_2_. At the *Clostridium* species level, the relative abundance of common autotrophic strains, such as *C. carboxidivorans, C. autoethanogenum, C. ragsdalei* and *C. ljungdahlii*, did not show much difference (Table 3). Liu et al. (2020) investigated the microbial community for 5 g/L glucose and syngas co-fermentation using the inoculum from a mesophilic anaerobic reactor. *Clostridium* spp. were enriched with a low relative abundance, and with the presence of mainly *Clostridium formicoaceticum.* However, butyrate was detected during the syngas and glucose co-fermentation, but not in the single glucose fermentation and hexanoic acid was not reported in their study.

## 5 Conclusion

The exogenous supply of 3.2 g/L butyric acid resulted in the production of 1.9 g/L butanol, while in the presence of exogenous acetic acid and ethanol, butanol production was enhanced to 2.6 g/L by an enriched sludge with a high abundance of *Clostridium* spp. using CO as the gaseous substrate. The presence of acetate and ethanol enhanced the butyric acid conversion efficiency compared to exogenous butyric acid. When increasing the exogenous butyric acid concentration to 14 g/L, the highest butanol concentration (3.4 g/L) was produced. Butanol accumulation was significantly enhanced up to 2.4 fold when adding CO after endogenous butyric acid production from glucose compared to when adding CO initially with glucose (co-fermentation), from which butanol was not efficiently produced with a similar amount of endogenous butyric acid production. The *Clostridia* and *Bacilli* class were dominantly enriched after glucose fermentation and at the end of the incubation. There is no big change in microbial community composition when comparing the end enriched sludge in the Glucose + CO and Glucose + N_2_ incubations.

## Data Availability

The original contributions presented in the study are included in the article/Supplementary Materials, further inquiries can be directed to the corresponding author.
